# Research Note: Role of darkling beetles (*Alphitobius diaperinus*) and litter in spreading and maintaining *Salmonella* Enteritidis and *Campylobacter jejuni* in chicken flocks

**DOI:** 10.1016/j.psj.2023.103061

**Published:** 2023-08-28

**Authors:** Subarna Barua, Matthew Bailey, Kevin Zhong, Nneka Iduu, Teresa Dormitorio, Kenneth Macklin, Dianna Bourassa, Stuart Price, Ruediger Hauck, James Krehling, Steven Kitchens, Constantinos Kyriakis, R. Jeff Buhr, Chengming Wang

**Affiliations:** ⁎ Department of Pathobiology, College of Veterinary Medicine, Auburn University, Auburn, AL, USA; † Department of Poultry Science, Auburn University, Auburn, AL, USA; ‡ Department of Poultry Science, Mississippi State University, Starkville, AL, USA; § PMSPRU, USNPRC, USDA-ARS, Athens, GA, USA

**Keywords:** darkling beetles (*Alphitobius diaperinus*), poultry litter, *Salmonella* Enteritidis, *Campylobacter jejuni*

## Abstract

*Salmonella* and *Campylobacter* are common foodborne pathogens in chickens, but their persistence mechanisms within flocks are not fully understood. In this study, 4 groups of SPF Leghorn chickens (*n* = 50) were orally inoculated with 10^8^*Salmonella* Enteritidis and 10^8^*Campylobacter jejuni*, housed in BSL-2 rooms inside containers with autoclaved bedding and beetles (*n* = 200). Phase I (wk 1–3): the infected chickens remained in the containers and were then euthanized while beetles and litter remained in the container (group A), beetles were removed and litter remained in the container (group B), beetles remained and litter was removed (group C), and beetles and litter were removed (group D). Phase II (wk 5–7): autoclaved bedding was added to containers in groups C and D, and new SPF chickens (*n* = 50) were introduced and kept. Phase III (wk 8–20): all chickens were euthanized, and the litter and/or beetles remained in the containers for 17 wk. The prevalence of *Salmonella* Enteritidis and *Campylobacter* was significantly higher when detected by PCR compared to culture. In phase II, when infected chickens were removed and new chickens were introduced, 1 fecal sample in group B and 3 litter samples in groups B and C were found positive for *Salmonella* Enteritidis, and *Campylobacter* was still detected in groups A, B, and C litter samples, but not in beetles. In phase III, when all chickens were removed, *Salmonella* Enteritidis was identified in beetle samples from group A and the litter samples of all tested groups A, B, and C, and *C. jejuni* was positive in litter samples from groups A and B but not in the beetle. Sixty-nine days after removing infected chickens, culturable *Salmonella* was still found in beetles. *Salmonella* and *Campylobacter* were detectable in litter up to 127 d after removing infected chickens. This study highlights the transmission of *Salmonella* and *Campylobacter* via beetles and litter to new flocks in successive rearing cycles. Intensive control programs should target insect exclusion and implement strict poultry litter management or litter changes between flocks.

## INTRODUCTION

The poultry industry, specifically broiler chicken, has been recognized as the fastest-growing segment, with a significantly increased market share in the United States. However, associated with this growth, the occurrence of foodborne illnesses caused by foodborne pathogens such as *Salmonella* spp. and *Campylobacter* spp. remains a significant concern within the US food industry.

Each year, *Salmonella* infections result in over 1 million cases and 420 deaths, contributing to approximately $4.4 billion in medical costs (CDC, 2023). Another pathogen of considerable food safety concern is *Campylobacter*; as reported by the USDA, even a small dose of 500 bacterial cells can lead to illness (USDA, 2013). These bacteria can enter the alimentary tract of birds and establish colonization through various sources, including litter (both fresh and reused), feed, water, pests (such as darkling beetles), and the surrounding environment.

The reused litter, in particular, can contain microorganisms shed by previous flocks, and since broiler chickens tend to scratch and consume litter, this can be a significant source of microorganisms. Litter can expose chicks to enteric pathogens such as *Salmonella* Enteritidis and *C. jejuni*, which they may not be immunologically mature enough to handle or defend against (Dunn et al., 2022). Proper litter management is crucial before, during, and after each poultry production cycle, especially in today's “No Antibiotics Ever” practices. This management is vital for reducing the transmission of these bacteria between flocks.

The darkling beetle (*Alphitobius diaperinus*), also known as the litter beetle, is a resilient pest commonly found in poultry houses. It is a scavenger arthropod and the most abundant pest in such environments. Both adult beetles and their larvae can survive within flocks by consuming chicken feed, feces, and even dead birds. Darkling beetles have been identified as significant vectors for several pathogenic bacteria affecting poultry, including *E. coli, Campylobacter*, and *Salmonella* Enteritidis (Donoso et al., 2020).

Recognizing the significance of litter and darkling beetles as vectors for foodborne pathogens is crucial in mitigating bacterial contamination during the live production phase of broilers. This understanding can aid in reducing the prevalence of these pathogens throughout preharvest, meat processing, and, ultimately, the consumer level. In this study, we conducted a systematic investigation to compare the role of darkling beetles and litter in transmitting the 2 most prevalent enteric pathogens, *S. enterica* Enteritidis and *C. jejuni*, between consecutive flocks housed in the same poultry facility.

## MATERIALS AND METHODS

### Bacterial Strains

The *Salmonella* Enteritidis strain used in this study was a nalidixic acid-resistant marker strain. Additionally, the *C. jejuni* strain employed in the research was a ciprofloxacin-resistant marker strain. Dr. Kenneth Macklin graciously provided both isolates.

### Insects

Adult black lesser mealworms (*Alphitobius diaperinus*) were obtained from a laboratory-bred colony maintained at Dr. Ruediger Hauck's laboratory, Auburn University. Before the start of all experiments, these beetles were tested and confirmed as *Salmonella* and *Campylobacter* negative by PCRs as described below.

### SPF Leghorn Layer Flocks

Specific pathogen-free (**SPF**) Leghorn chickens, aged 3 wk, were purchased from Charles River (Norwitch, CT). They were subsequently housed in isolation rooms with BSL-2 containment at the Sugg Isolation Facility, College of Veterinary Medicine, Auburn University. The chickens were placed in bulk high-density polyethylene containers (Uline, WI) with dimensions of 45 × 45 × 33 inches, while autoclaved pine shaving bedding was included. Throughout the study, the chickens were closely monitored 2 to 3 times daily to ensure their welfare. Adequate feed and water were provided ad libitum. This research project was approved by the Auburn University Institutional Biosafety Committee (BUA # 981) and the University Institutional Animal Care and Use Committee (IACUC # 2022-5076).

### Inoculation of Chickens With Bacteria

*Salmonella* Enteritidis and *C. jejuni* strains were grown individually and mixed with PBS to create a concentration of 10^8^ CFU per mL for both *Salmonella* Enteritidis and *C. jejuni*. All chickens received a single oral gavage of 1 mL of the bacterial cocktail using a tuberculin syringe.

### Experimental Design

This study allocated 50 three-wk-old SPF chickens to 4 groups and housed them in separate isolation rooms. The chicken distribution during phases I and II was as follows: group A (*n* = 13), group B (*n* = 13), group C (*n* = 12), and group D (*n* = 12). On d 0, all chickens were orally inoculated with *Salmonella* Enteritidis and *C. jejuni*, while 200 adult beetles were introduced into the litter of each container on the same day.

Following the euthanization of the chickens at the end of phase I at wk 3, the treatment of beetles and litter differed among the groups. In group A, the beetles and used litter were retained in the container. In group B, stackable mealworm sifting trays were used to trap beetles from the litter. In this group, only the beetles were removed, leaving the used litter in the container. Group C maintained the beetles, but the litter was removed, the container was cleaned and disinfected using Virocid disinfectant (Cid Lines, Ieper, Belgium), and then replaced with new autoclaved bedding. In group D, the beetles and the litter were removed; the container was cleaned and disinfected using Virocid disinfectant (Cid Lines, Ieper, Belgium) and fresh autoclaved bedding was provided. After the 1-wk interval after phase I, a new batch of SPF chickens (*n* = 50) was introduced into each container of the 4 groups for phase II. The chickens were maintained for 3 wk (wk 5–7) before euthanizing.

In phase III, the litter from groups A, B, and C and the beetles from groups A and C remained in the containers for an additional 17 wk (wk 8–20). Group D was excluded from phase III.

### Sampling

Fresh fecal swabs from each bird during wk 1 to 3 (phase I) and wk 5 to 7 (phase II) were collected to assess the *Salmonella* Enteritidis and *C. jejuni* status in the chicken flocks. The swabs were immediately placed in a 15-mL centrifuge tube containing 2 mL of PBS and transported to the laboratory for analysis.

For beetle sampling, 10 beetles were randomly collected from each container in phases I and II and from groups A, B, and C in phase III. The beetles were placed in centrifuge tubes, transported to the laboratory on sampling day, and homogenized with 1 mL of PBS using 4 zirconia beads in a Precellys 24 lysis and homogenization instrument (Bertin Instruments, France).

Litter samples were collected from each container of the 4 groups in all 3 phases (phases I–III). The samples were placed in Ziploc bags and transported to the laboratory. After thoroughly mixing the sample, 1 g of litter was weighed and vortexed in 10 mL of PBS. Sampling frequency was weekly during phases I and II, while in phase III, samples were collected on a monthly basis to assess the presence of *Salmonella* Enteritidis and *C. jejuni*.

### Isolation of *Salmonella* Enteritidis and *C. Jejuni*

The collected and processed samples (500 µL) from chicken fecal swabs, beetle homogenates, and litter were directly spread on selective media to isolate *Salmonella* Enteritidis. Xylose lysine agar (XLT4 Agar Base, BD) supplemented with tergitol 4 (XLT4 Agar Supplement, BD) and nalidixic acid antibiotic (35 ppm) was used. These plates were incubated at 37°C for 24 to 48 h, and colonies with black or black-centered appearance (indicating H_2_S production) were considered potential *Salmonella* Enteritidis isolates.

For the bacteriological examination of *Campylobacter* spp., 500 µL of the processed samples from fecal swabs, beetle homogenates, and litter were plated directly onto Campy Cefex agar (Neogen, MI) supplemented with horse blood and ciprofloxacin (2 ppm). Characteristic small, mucoid, and grayish colonies were observed to identify *Campylobacter*.

### Quantification of *Salmonella* Enteritidis and *C. Jejuni* by PCRs

The extraction of total nucleic acids from the collected samples (200 µL) was performed using the High Pure PCR Template Preparation Kit (Roche Diagnostics, Indianapolis, IN) following the manufacturer's instructions. The *Salmonella* Enteritidis and *C. jejuni* PCRs were conducted on a Roche light cycler 480 II systems (Roche Molecular Biochemicals, Indianapolis, IN) as previously described (Zhang et al., 2013).

Fluorescence Resonance Energy Transfer (**FRET**)-PCR was performed for the *Salmonella* Enteritidis quantification that amplifies a 216-bp target. This PCR targets the tetrathionate reductase response regulator (***ttrR***) gene, a highly conserved gene located within the *Salmonella* pathogenicity island 2. The positions of primers and probes (synthesized by Integrated DNA Technologies, Coralville, IA) are as follows: forward primer: 5′-GATGTTYCTTAGCGCYTTACAGGC-3′; reverse primer: 5′-CCGACMGCGTAATATTTGGCTGAC-3′; anchor probe: 5′-ATACGCTTTCCGGCACGGCAAT-6-FAM-3′; reporter probe: 5′-LCRED640-CGTCRGTGGATTWCCGTCGCCCT-Phosphate-3′ (Zhang et al., 2013).

To quantify *C. jejuni* in extracted samples, a TaqMan real-time PCR was performed in a 20 µL volume. This PCR targets a virulence gene known as *mapA* gene using the following primers and probes: forward primer 5′-TGCTCAAGTTAATCAAATTTC-3′, reverse primer 5′-CCCTTTAATCTTTGCTTCA-3′ and probe 5′-ROX ACCACCAGGACTTTCACAAGAACT BHQ2-3′ (**ROX** = 6-carboxy-X-rhodamine; **BHQ2** = black hole quencher 2) with an 82-bp amplicon size as described (Liu et al., 2019).

Quantitative standards for *Salmonella* Enteritidis and *C. jejuni* were commercially synthesized as target DNA cloned into an expression plasmid. The standards included 5 concentrations: 10,000, 1,000, 100, 10, and 1 copies per reaction mixture. These quantitative standards were run alongside each PCR assay, and negative control of sterile water was included. The PCR products were validated by sequencing at ELIM Biopharmaceuticals (Hayward, CA).

### Statistical Analysis

All statistical analyses were performed using Statistica 7.0 software package (StatSoft, Inc., Tulsa, OK). A chi-square test was performed to compare the prevalence of *Salmonella* Enteritidis and *C. jejuni*, and the detection rates of *Salmonella* or *Campylobacter* in different types of samples. A difference at *P*  ≤  0.05 was considered significant.

## RESULTS AND DISCUSSION

The prevalence of *Salmonella* Enteritidis was significantly higher when detected by PCR compared to culture in fecal swabs (59/300 vs. 20/300; *P* < 10^−4^) and in litter (23/39 vs. 10/39; *P* = 0.003). Similarly, the positivity of *Campylobacter* was also significantly higher when detected by PCR as compared to culture in fecal swabs (131/300 vs. 106/300; *P* = 0.04) and in the litter (24/39 vs. 7/39; *P* = 0.0001). The detection rates for both bacteria were not significantly different in beetles. The specificity of the PCRs used in this study was ensured by validated primers and probes designs and DNA sequencing. The detection limit for the PCRs used in this study was 1 copy of the target gene per reaction (equivalent to 200 copies/fecal swab, 10 copies/beetle, 1,000 copies/g of litter). Therefore, the analysis of bacterial prevalence was based on the detection of *Salmonella* and *Campylobacter* PCRs.

In phase I, when the infected chickens remained in the container, both pathogens were identified by PCRs in fecal swabs, beetles, and litter of all 4 groups (Tables 1 and 2). The positivity of *C. jejuni* (128/150) was significantly higher than *Salmonella* Enteritidis (58/150) in fecal swabs (chi-square, *P* < 10^−4^). Additionally, the detection rate of *Campylobacter* was significantly higher in fecal swabs than in beetles (128/150 vs. 7/16, *P* < 10^−4^) (Table 2).

**Table 1:** Prevalence of *Salmonella* identified by culture and PCR in chicken fecal samples, beetles, and litter.

			Positive samples by culture				Number and copies of positive samples by qPCR (1)			
Sample	Phase	Collection	A	B	C	D	A	B	C	D
Fecal samples	I	1	0	0	0	3	8; 8.12 × 10^4^	7; 1.13 × 10^5^	5; 2.92 × 10^5^	3; 2.90 × 10^3^
		2	0	0	1	3	5; 3.68 × 10^4^	9; 1.62 × 10^4^	4; 3.97 × 10^4^	2; 2.32 × 10^4^
		3	2	2	0	0	4; 5.56 × 10^4^	6; 9.57 × 10^4^	3; 9.80 × 10^4^	2; 3.91 × 10^4^
	II	1	0	3	0	0	0	0	0	0
		2	0	4	0	0	0	1; 4.37 × 10^2^	0	0
		3	0	2	0	0	0	0	0	0
Beetles	I	1	0	0	1	0	1; 3.61 × 10^3^	0	1; 7.00 × 10^5^	1; 1.67 × 10^4^
		2	1	1	1	0	1; 9.45 × 10^3^	1; 3.33 × 10^5^	1; 2.47 × 10^5^	1; 3.42 × 10^5^
		3	0	0	1	1	0	0	1; 1.19 × 10^3^	1; 2.95 × 10^3^
	II	1	0	N/A^2^	1	N/A	0	N/A	0	N/A
		2	0		0		0		0	
		3	0		0		0		0	
	III	1	0		0		0		0	
		2	0		0		1; 8.45 × 10^2^		0	
		3	0		0		0		0	
		4	0		0		0		0	
		5	0		0		0		0	
Litter	I	1	0	0	1	0	1; 7.35 × 10^2^	1; 6.80 × 10^5^	1; 3.62 × 10^7^	1; 1.95 × 10^4^
		2	1	1	1	0	1; 2.70 × 10^5^	1; 4.24 × 10^6^	1; 7.25 × 10^6^	1; 2.00 × 10^6^
		3	1	1	1	1	1; 8.02 × 10^4^	1; 7.90 × 10^4^	1; 2.63 × 10^6^	1; 4.03 × 10^5^
	II	1	1	1	0	0	0	1; 1.30 × 10^4^	0	0
		2	0	0	0	0	0	0	0	0
		3	0	0	0	0	0	1; 1.34 × 10^4^	1; 2.13 × 10^4^	0
	III	1	0	0	0	N/A	1; 3.34 × 10^4^	1; 4.74 × 10^4^	0	N/A
		2	0	0	0		0	0	1; 2.24 × 10^3^	
		3	0	0	0		0	0	0	
		4	0	0	0		1; 4.51 × 10^4^	1; 3.32 × 10^3^	0	
		5	0	0	1		1; 1.17 × 10^5^	1; 7.75 × 10^5^	1; 1.25 × 10^5^	

1 Copy number of *Salmonella* by PCR is shown per fecal swab, 10 beetles, or 1 g litter.

2 NA means not applicable, as beetles were unavailable at this experiment stage.

**Table 2:** Prevalence of *Campylobacter* identified by culture and PCR in chicken fecal samples, beetles, and litter.

			Positive samples by culture				Number and copies of positive samples by PCR (1)			
Sample	Phase	Collection	A	B	C	D	A	B	C	D
Fecal samples	I	1	2	4	5	5	10; 1.25 × 10^3^	11; 1.16 × 10^5^	6; 1.78 × 10^3^	5; 7.50 × 10^2^
		2	5	1	2	2	13; 2.84 × 10^4^	13; 1.24 × 10^3^	9; 2.82 × 10^3^	11; 1.83 × 10^4^
		3	13	13	11	12	13; 1.67 × 10^6^	13; 1.70 × 10^5^	12; 4.20 × 10^5^	12; 7.06 × 10^5^
	II	1	1	6	3	0	1; 7.5 × 10^2^	1; 7.50 × 10^2^	0	0
		2	3	6	1	0	0	0	0	0
		3	3	5	3	0	0	0	0	0
Beetles^2^	I	1	0	0	0	0	0	0	0	0
		2	0	1	0	1	1; 7.90 × 10^2^	1; 5.93 × 10^3^	1; 2.99 × 10^2^	1; 1.05 × 10^2^
		3	0	0	0	0	1; 1.00 × 10^2^	0	1; 1.00 × 10^2^	1; 1.00 × 10^2^
	II	1	0	N/A						
	0	N/A	0	N/A	0	N/A				
		2	0		0		0		0	
		3	0		0		0		0	
	III	1	0		0		0		0	
		2	0		0		0		0	
		3	0		0		0		0	
		4	0		0		0		0	
		5	0		0		0		0	
Litter	I	1	0	0	0	0	1; 7.50 × 10^2^	1; 7.50 × 10^2^	0	0
		2	0	0	1	0	1; 1.01 × 10^4^	1; 2.25 × 10^4^	1; 1.08 × 10^4^	1; 2.34 × 10^4^
		3	1	0	1	0	1; 7.33 × 10^3^	1; 2.86 × 10^3^	1; 1.63 × 10^4^	1; 3.15 × 10^3^
	II	1	1	1	0	0	1; 7.50 × 10^2^	1; 7.50 × 10^2^	1; 7.50 × 10^2^	0
		2	1	0	0	0	1; 1.05 × 10^3^	1; 1.05 × 10^3^	0	0
		3	1	1	0	0	1; 1.05 × 10^3^	1; 1.05 × 10^3^	0	0
	III	1	0	0	0	N/A	1; 7.50 × 10^2^	1; 7.50 × 10^2^	0	N/A
		2	0	0	0		1; 1.10 × 10^4^	0	0	
		3	0	0	0		1; 7.50 × 10^2^	0	0	
		4	0	0	0		1; 7.50 × 10^2^	0	0	
		5	0	0	1		1; 1.92 × 10^5^	1; 7.20 × 10^2^	0	

1 Copy number of *Campylobacter* by PCR is shown per fecal swab, 10 beetles, or 1 g litter.

2 NA means not applicable, as beetles were unavailable at this experiment stage.

Moving to phase II, when infected chickens were removed and new chickens were introduced, only 1 fecal sample in group B and 3 litter samples in groups B and C were found positive for *Salmonella* Enteritidis. In this phase, the infection rate of *C. jejuni* also significantly decreased in fecal swabs and was found positive in groups A and B only. However, *Campylobacter* was still detected in groups A, B, and C litter samples, but not in beetles.

Transitioning to phase III, when all chickens were removed, *Salmonella* Enteritidis was identified in beetle samples from group A and the litter samples of all tested groups A, B, and C (Table 1). In this phase, *C. jejuni* was positive in litter samples from groups A and B but not in the beetle samples (Table 2). The copy numbers of *Salmonella* and *Campylobacter* were found to be higher in phase I than in phases II and/or III in the assayed samples (Tables 1 and 2).

While *Salmonella* Enteritidis was detected in beetles in all 4 groups in phase I, this pathogen was only detected in group A of phase III. Culture and PCR with DNA sequencing confirmed that viable *Salmonella* Enteritidis was identified in beetle 69 d after the infected chickens were removed. *Campylobacter* was not detected in beetles of all groups in phases II and III after the infected chickens were removed.

The detection rates in the litter for both pathogens were higher than in beetles after the infected chickens were removed. In litter, 11 of 24 litter samples (45.8%) were positive for *Salmonella* Enteritidis, and 14/24 (58.3%) were positive for *C. jejuni*. Viable *Salmonella* Enteritidis and *C. jejuni* remained detectable for up to 127 d, the end of the experiment, in litter after the infected chickens were removed.

The prevalence pattern of *C. jejuni* in chickens in this study aligns with findings reported in the existing literature. It has been documented that once introduced, *C. jejuni* can rapidly spread horizontally throughout a broiler facility (Bailey et al., 2022). In our study, *C. jejuni* positivity ranged from 42 to 85% in the first week across 4 groups, reaching 100% prevalence in all chickens by the third week until the euthanization of all infected chickens. The spread of *C. jejuni* also extended to the environment, as evidenced by the detection of this pathogen in both beetles and litter across all groups during this period.

In our investigation, beetles were found to be positive for both *Salmonella* Enteritidis and *C. jejuni* in the presence of infected chickens (phase I of this study). This finding is consistent with other studies that have identified beetles as potential carriers of *Salmonella* and *Campylobacter* (Donoso et al., 2020). Significantly, 69 d after the infected chickens were removed, culture and PCR confirmed the presence of viable *Salmonella* in beetles.

In our study, we incorporated a 1-wk period between rearing cycles, a common practice in many countries. During this process, removing old litter generally leads to the elimination from the house of many litter beetles. However, beetles likely hide in the insulation material or crevices of the buildings. To simulate the potential role of these contaminated beetles in pathogen transmission, we designed group C where the old litter was removed, but the beetles were retained in the container.

In group C, after removing the infected chickens, we detected *Salmonella* and *Campylobacter* in the litter samples but not in the beetles. Based on the experimental design, it is highly likely that the contaminated beetles were the source of *Salmonella* and *Campylobacter* found in the litter samples. It is worth noting that beetles were tested weekly in phase II and monthly in phase III of the study. The low sampling frequency and potentially low bacterial burden in the beetles may have contributed to the false-negative results in their beetle detection.

Previous studies have also indicated that beetles and their larvae are only positive for *Campylobacter* when the chickens themselves are positive (Bates et al., 2004). However, despite not being directly *Campylobacter*-positive, beetles can still contribute to the transmission of *Campylobacter* within chickens (Bates et al., 2004).

During our investigation, we observed that litter samples collected during the presence of infected chickens, noninfected chickens, and even in the absence of chickens tested positive for *S.* Enteritidis, *C. jejuni*, or both. These findings align with earlier studies that have identified litter as a vehicle for transmitting *Salmonella* (Dunn et al., 2022) and *Campylobacter* (Bailey et al., 2022). In group B, where only potentially contaminated litter remained, we found 1 chicken positive for S. Enteritidis, suggesting the transmission of the pathogens between consecutive broiler flocks through *Salmonella*-contaminated litter. Furthermore, our study demonstrated that pathogens could persist in the litter for up to 127 d within the study period, potentially serving as a continuous source of these pathogens in subsequent flocks if the litter is reused without proper treatment. Kelley et al. reported the presence of these pathogens in the litter for up to 40 d of reutilization (Kelley et al., 1994).

During our investigation, we observed a significantly higher detection rate of *S*. Enteritidis and *C. jejuni* using PCR compared to culture-based methods in fecal swabs, beetles, and litter samples. This finding indicates that the culture-based methods for detecting these pathogens, such as *S*. Enteritidis and *C. jejuni*, may not be sufficiently sensitive. One possible factor that could contribute to the low detection rate of *Salmonella* and *C. jejuni* in culture-based methods for beetles and litter samples is the potential formation of a viable but nonculturable (**VBNC**) state of the cells (Gu et al., 2019).

One limitation of this this study was not to differentiate external and internal *Salmonella* and *Campylobacter* in the beetles. Likely, the pathogens are not part of gut microbiome of beetles and just on their external surface. The future study could consider sanitizing the external surface (exoskeleton) of the beetles before detecting the pathogens.

Another limitation of our study is the relatively small number of beetles (*n* = 10) and the limited number of samples collected at each collection time. This small sample size and low sampling frequency may not fully reflect the real prevalence of the pathogens in the beetles and litter. In this study, the direct plating for prevalence of Salmonella was performed, and the more sensitive *Salmonella* culture method with pre-enrichment on more samples shall be used on the future studies.

This study suggested that *Salmonella* and *Campylobacter* can be transmitted via beetles and litter to new flocks in successive rearing cycles. Implementing intensive control programs to exclude poultry insects and practicing proper poultry litter management between flocks will help reduce the spread of foodborne pathogens between rearing cycles in broiler houses.
